# Chronic Stress, Diabetes, and Cardiovascular Disease: Epidemiologic Evidence, Pathogenetic Insights, and Therapeutic Strategies

**DOI:** 10.7759/cureus.102108

**Published:** 2026-01-22

**Authors:** Mahri Hatamova, Mario Rizk, Keston Rattan, Andrew Gaballa, Samy I. McFarlane

**Affiliations:** 1 Department of Medicine, State University of New York Downstate Health Sciences University, Brooklyn, USA

**Keywords:** cardiovascular disease, coronary heart disease, diabetes, epidemiology, pathogenesis, psychological distress, stress management, stroke

## Abstract

Chronic stress has been shown in cross-sectional as well as longitudinal studies to be strongly associated with diabetes, hypertension, and cardiovascular disease (CVD). These studies have used various methods of stress assessment. Proposed pathogenetic mechanisms linking psychological distress with diabetes and CVD are complex and include stimulation of the hypothalamic-pituitary-adrenal axis with excess cortisol release, as well as stimulation of the renin-angiotensin-aldosterone system, resulting in elevated blood pressure, in addition to an altered immune system, increased inflammation, and oxidative stress, among others. In this review, we provide the reader with cutting-edge information on the current evidence for the association between chronic psychological stress, diabetes, and CVD, highlighting the major studies addressing this important topic. We also provide a detailed mechanistic overview of the pathogenesis of diabetes and CVD in psychologically stressed individuals, including experimental and laboratory evidence. We finally discuss the major evidence-based therapeutic interventions for psychological stress and their effects on CVD risk reduction, with the aim of presenting these therapeutic options as potentially effective ways to implement in clinical practice.

## Introduction and background

Psychological distress has been shown to be strongly associated with diabetes risk as well as poor glycemic control, among diabetic patients in various populations [1-5]. Chronic stress has also been implicated as a risk factor for hypertension, stroke, and cardiovascular disease (CVD) [3,6-15]. Various scales and assessment tools have been utilized in the identification of psychological stress among different populations (Table 1) [16-30].

**Table 1:** Overview of validated instruments used to assess chronic psychological stress in epidemiological research The table includes each scale’s full name, year of development, core domains assessed, structure (e.g., self-report, interview-based), and typical population usage. This summary illustrates the diversity of tools employed in population-level studies and their methodological evolution across decades CES-D: Center for Epidemiologic Studies Depression Scale; CARDIA: Coronary Artery Risk Development in Young Adults; NHANES: National Health and Nutrition Examination Survey; MIDUS: Midlife in the United States; NHIS: National Health Interview Survey

Scale name	Developer/year	Focus	Used in
CES-D Scale	Radloff [18], 1977	Depressive symptoms	Population mental health studies
Life Events and Difficulties Schedule	Brown and Harris [19], 1978	Contextual life events and chronic stress	Longitudinal studies
Job Content Questionnaire	Karasek [23], 1979	Job demands, control, support	Occupational health research
General Health Questionnaire	Goldberg and Hillier [20], 1979	Psychological strain	UK and global surveys
Perceived Stress Scale	Cohen et al. [22], 1983	Subjective stress perception	CARDIA, NHANES
Daily Hassles and Uplifts Scale	DeLongis et al. [21], 1988	Minor daily irritants and positive events	Stress process studies
Chronic Burden Scale	Turner et al. [25], 1995	Ongoing stressors (health, finances, etc.)	CARDIA, MIDUS
Effort Reward Imbalance	Siegrist [24], 1996	Work stress via imbalance	European cohort studies
Trier Inventory for Chronic Stress	Schulz and Schlotz [26], 1999	Chronic stress across domains	German cohort studies
Kessler Distress Scale (K10/K6)	Kessler et al. [27], 2002	Nonspecific psychological distress	NHIS, WHO surveys
Stress Overload Scale	Amirkhan [28], 2003	Demand vs. coping capacity	Community stress research

**Figure 1:**
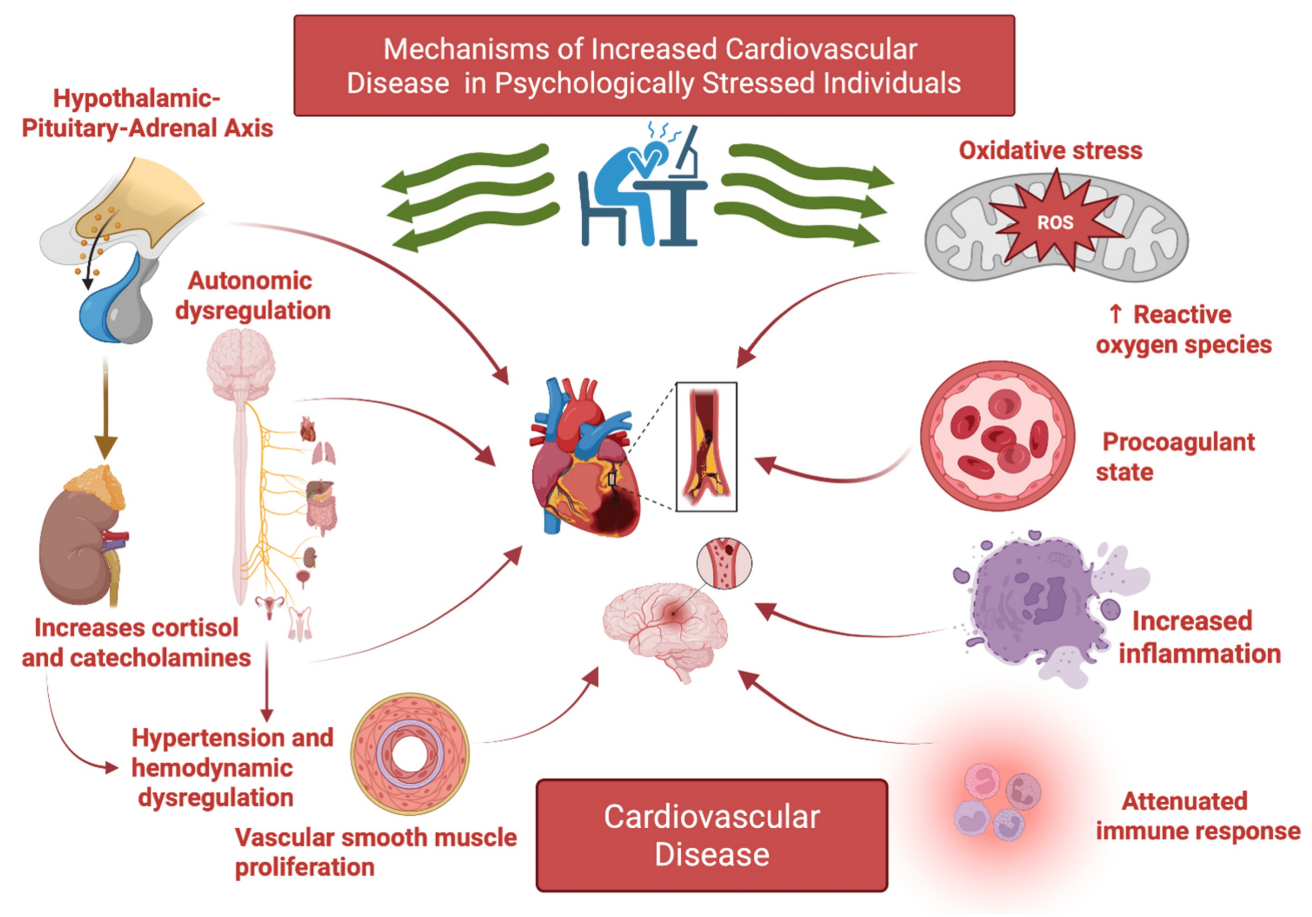
Mechanistic pathways of increased CVD in psychologically stressed individuals ROS: reactive oxygen species; CVD: cardiovascular disease Image credit: This is an original image created by the author Mahri Hatamova Source: [6-15,31,32]

Proposed pathogenetic mechanisms for diabetes and cardiovascular dysfunction associated with chronic psychological stress include neuroendocrine dysregulation, such as stimulation of the hypothalamic-pituitary-adrenal (HPA) axis, stimulation of the renin-angiotensin-aldosterone system (RAAS), increased inflammation, a procoagulant state, and an attenuated immune response (Figure 1) [31,32].

**Figure 2:**
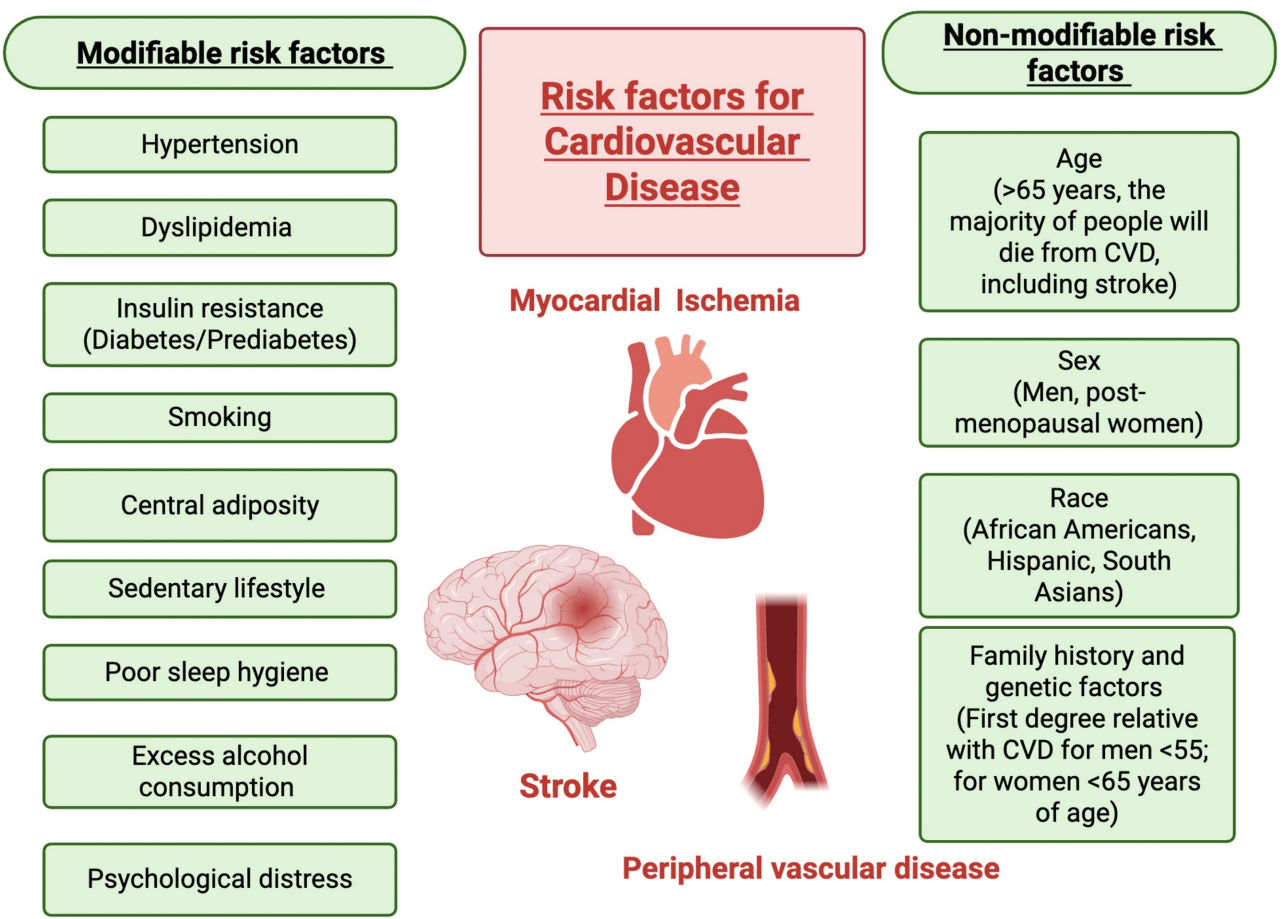
Modifiable and nonmodifiable risk factors of cardiovascular disease Image credit: This is an original image created by the author Mahri Hatamova Source: [32-35]

Throughout the years, CVD risk factors have been identified and periodically updated. These factors are traditionally classified as modifiable and nonmodifiable ones (Figure 2) [33].

Despite identification of risk factors and the monumental healthcare resources utilized, control of CVD remains largely suboptimal, and CVD remains one of the major causes of morbidity and mortality in the United States and around the world [34,35]. Therefore, identification of additional modifiable risk factors for CVD is of major importance; psychological distress appears to be an attractive and potentially modifiable therapeutic target for CVD risk reduction [32].

In this narrative review, we present the epidemiological evidence for the association between psychological distress, diabetes, and CVD. We also discuss the various tools and the context in which they were used to assess psychological distress in these studies. We highlight the putative mechanisms of vascular injury and insulin resistance in psychologically distressed individuals, and finally, we provide the reader with updated information on the various interventions used to address psychological distress [17,36-40] that might be implemented in the daily clinical practice.

## Review

Epidemiological evidence of the association of chronic stress, diabetes, and CVD

Assessment of Chronic Psychological Stress in Epidemiological Research

Accurate assessment of chronic psychological stress is fundamental to understanding its role in population health, particularly in relation to CVD and diabetes [16,17]. Over the past several decades, researchers have developed a variety of instruments to assess either sustained exposure to stressors or psychological states reflecting long-term strain (Table 1) [18-28]. One of the earliest and most widely used tools is the Center for Epidemiologic Studies Depression Scale, introduced by Radloff in 1977. Although originally designed to assess depressive symptoms, it is often used as a proxy for emotional stress in epidemiological research [18]. Around the same time, the Life Events and Difficulties Schedule brought an important methodological advance by capturing the severity and chronicity of stressors through contextualized interviews [19].

As research attention shifted toward more subtle and persistent forms of stress, tools like the General Health Questionnaire (GHQ) and the Daily Hassles and Uplifts Scale were introduced. The GHQ screens for general psychological strain, while the Daily Hassles scale captures the cumulative impact of minor but frequent stressors, offering a more granular view of ongoing stress [20,21]. The development of the Perceived Stress Scale (PSS) in 1983 marked a turning point, providing a validated measure of how individuals perceive and appraise stress in their daily lives [22].

Stress in occupational settings prompted the creation of domain-specific tools. The Job Content Questionnaire, based on the demand-control model [23], and the Effort-Reward Imbalance scale assess chronic stress arising from work environments, where high demands and low rewards or autonomy contribute to long-term strain [24]. To capture broader life challenges, the Chronic Burden Scale was introduced to assess enduring stress in areas such as health, relationships, and finances, which is especially relevant in long-term cohort studies [25].

The TICS, developed in the late 1990s, built on these frameworks with a multidomain inventory rooted in psychobiological models [26]. More recently, the Kessler Psychological Distress Scales (K10/K6) were developed for large-scale surveys, providing brief yet reliable tools to monitor persistent psychological distress across populations. The 10-item version offered robust assessment, while the six-item version (K6) was developed for national health surveys such as the National Health Interview Survey and WHO studies, balancing brevity with high specificity [27]. The Stress Overload Scale, developed in 2003, addresses both the magnitude and duration of perceived demands, offering a dynamic, threshold-based model for assessing chronic stress [28].

Collectively, these instruments reflect the evolution of stress assessment from broad emotional states to domain-specific burdens and multidimensional frameworks [18-28]. Their use in epidemiological studies has enabled consistent, scalable assessments of chronic psychological distress and its contribution to cardiometabolic risk [29,30].

Evidence in Cross-Sectional Studies

Assessment of chronic psychological stress in cross-sectional studies is typically conducted using validated self-report tools, such as the PSS and the Screening Scale of Chronic Stress (SSCS). The latter is a brief, self-report instrument designed to measure chronic stress burden in population-based and clinical research, derived from the longer Trier Inventory for Chronic Stress (TICS) [41-43]. Additionally, structured interviews and, less often, objective biomarkers such as hair cortisol, salivary cortisol, and heart rate variability (HRV) are used. These tools capture both subjective and physiological aspects of stress, although self-report measures may overlap with symptoms of depression and anxiety, and physiological markers provide complementary but not interchangeable information [41-43].

Cross-sectional studies have consistently demonstrated that chronic psychological stress is associated with increased prevalence and risk of both CVD and diabetes mellitus. Chronic stress burden is independently associated with higher prevalence of coronary heart disease (CHD), stroke, diabetes, and hypertension, even after adjustment for sociodemographic and behavioral confounders [43,44]. Both cumulative exposure to daily stressors and exposure to traumatic stress are linked to elevated CVD risk, with evidence indicating that work-related stress, perceived stress, and social isolation each contribute to increased incidence of CVD events [41,43-46]. The risk is not limited to adulthood; adverse childhood experiences and social disadvantage are also associated with higher levels of inflammation and metabolic risk factors later in life [41].

In the context of diabetes, higher levels of perceived chronic stress are linked to increased predicted risk of type 2 diabetes, impaired glucose control, insulin resistance, and increased inflammatory markers [42,47,48]. Chronic stress activates the HPA axis and the SNS, resulting in increased secretion of glucocorticoids and catecholamines. These neuroendocrine changes promote gluconeogenesis, suppress glucose uptake, increase lipolysis, and induce insulin resistance and inflammation, all of which contribute to the pathogenesis of type 2 diabetes [41,42,47,48]. Comorbid stress and depressive symptoms in individuals with diabetes are associated with a higher incidence of adverse cardiovascular outcomes, including stroke and cardiovascular death [47,48].

Mechanistically, chronic psychological distress leads to activation of neuroendocrine and inflammatory pathways, resulting in hypercoagulability, dyslipidemia, impaired glucose control, and endothelial dysfunction (Figure 1) [41,49,50]. These processes contribute to the development and progression of atherosclerosis and increase the risk of acute cardiovascular events. Chronic stress is also associated with increased sympathetic tone, decreased vagal tone, decreased HRV, and increased arterial stiffness, further exacerbating cardiovascular risk [41,46,49,50]. The cumulative burden of chronic stress, conceptualized as allostatic load, mediates the relationship between stressful life events and cardiometabolic multimorbidity [49,51].

While cross-sectional designs cannot establish causality, the robust associations observed across diverse populations and multiple indicators of stress highlight the importance of assessing and addressing chronic psychological stress as a modifiable risk factor in the prevention and management of both CVD and diabetes.

Evidence in Longitudinal Studies

Chronic stress is independently associated with an increased risk of both type 2 diabetes and CVD in multiple longitudinal studies. Chronic stress, whether from daily stressors, financial strain, or stressful life events, promotes dysregulation of the HPA axis and sympathetic nervous system (SNS), leading to increased glucocorticoid and catecholamine secretion, which in turn drives insulin resistance, hyperglycemia, and inflammation, thereby increasing the risk of type 2 diabetes and adverse cardiometabolic outcomes [47,48,52].

Longitudinal cohort studies consistently demonstrate that cumulative exposure to stressful life events is associated with a higher risk of developing type 2 diabetes (hazard ratios ranging from 1.05 to 1.23 per unit increase in stress exposure) and earlier age at diagnosis [52]. Prospective data, such as from the Australian Longitudinal Study on Women's Health, show that women with moderate to high perceived stress have more than a twofold increased risk of developing type 2 diabetes over 12 years, independent of traditional risk factors including obesity, hypertension, and physical inactivity [53].

Chronic stress also increases the risk of incident CVD, including CHD, stroke, myocardial infarction, heart failure, and cardiovascular mortality, with hazard ratios typically in the range of 1.2-1.4 for high stress exposure [41,54-56]. Large-scale analyses from the UK Biobank further demonstrate that chronic stress, particularly financial strain, is associated with significantly higher risks of stroke, CHD, myocardial infarction, and heart failure, with mediation analyses implicating body composition, lifestyle, immune function, and metabolic factors such as HbA1c [54].

Both cumulative daily stressors and traumatic stress exposures are associated with increased incident CVD risk. The American Heart Association (AHA), in its 2021 scientific statement, confirms that cumulative exposure to daily stressors as well as traumatic stress increases CVD risk, and meta-analyses of prospective studies have shown that work-related stress is associated with a 40% increased risk of incident CVD (relative risk [RR], 1.4; 95% CI, 1.2-1.8) and that high perceived stress is linked to a 27% increased risk of incident CHD and CHD mortality (RR, 1.27; 95% CI, 1.12-1.45) [41,57]. Social isolation and loneliness, common sources of stress, are associated with a 50% increased risk of CVD events [58].

In individuals with diabetes, comorbid chronic stress and depressive symptoms further elevate the risk of adverse cardiovascular outcomes, including stroke and cardiovascular death, with additive effects demonstrated in large prospective cohorts [58,59]. The accumulation of stressful life events is also associated with increased prevalence of cardiometabolic multimorbidity, mediated in part by allostatic load [60].

Mechanistic insights into the pathogenesis of insulin resistance and CVD in psychological distress

Neuroendocrine Dysfunction

**Figure 3:**
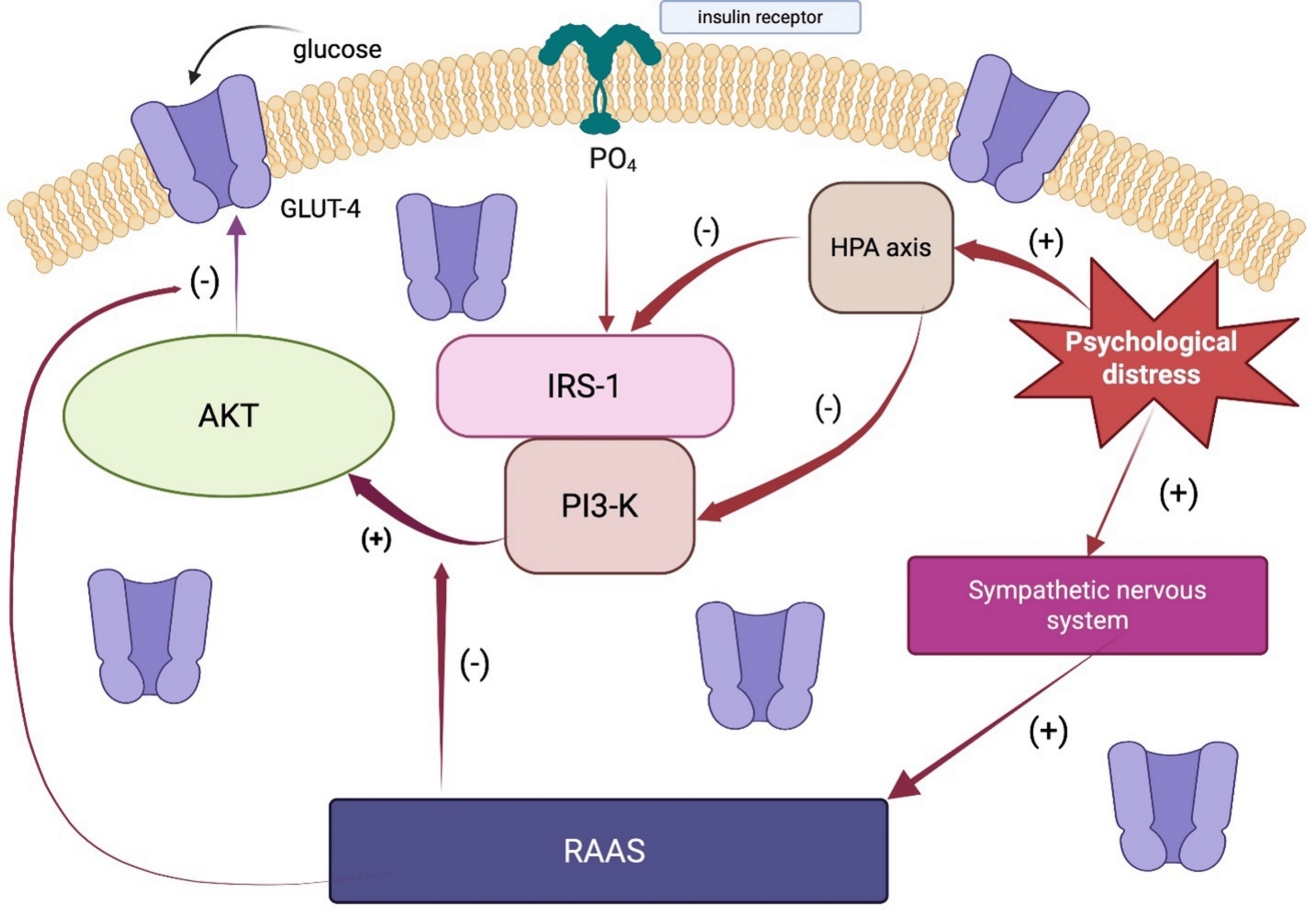
Stress-mediated impairment of insulin signaling Psychological distress leads to stimulation of the HPA axis, which inhibits IRS-1 and PI-3 kinase, interrupting the insulin-signaling pathway. Psychological distress also stimulates the sympathetic nervous system, stimulating the renin-angiotensin-aldosterone system. These mechanisms collectively inhibit the movement of GLUT-4 to the surface membranes of muscle and fat cells, thereby impeding insulin-mediated glucose uptake, creating a milieu of insulin resistance and metabolic dysregulation HPA: hypothalamic-pituitary-adrenal; IRS-1: insulin receptor substrate 1; PI3-K: phosphatidylinositol 3 kinase; RAAS: renin-angiotensin-aldosterone; GLUT-4: glucose transporter-4 Image credit: This is an original image created by the author Mahri Hatamova Source: [41,48,61-63]

Psychological distress in adults, such as chronic stress, depression, and anxiety, results in the activation of neuroendocrine changes that are central to the pathogenesis of insulin resistance and CVD. The primary neuroendocrine systems involved include the HPA axis, the SNS, and the RAAS. Activation of the HPS axis begins with hypothalamic release of corticotropin-releasing hormone, which stimulates pituitary secretion of adrenocorticotropic hormone (ACTH). ACTH then drives adrenal cortisol production. In chronic psychological distress, this axis becomes dysregulated, resulting in sustained elevations or loss of the diurnal patterns of cortisol secretion. Individuals with depression or chronic stress often exhibit a blunted cortisol awakening response in addition to a flattened diurnal cortisol curve, both of which have been found to be associated with increased insulin resistance and a higher risk of type 2 diabetes mellitus (T2DM) in adults (Figure 3) [48,61,62].

Flattening of the diurnal cortisol curve is also associated with a 1.5- to 2-fold increase in the risk of developing type 2 diabetes [61]. Sustained elevations in cortisol also promote hepatic gluconeogenesis, increase lipolysis, and suppress glucose uptake in skeletal muscle and adipose tissue, while also impairing pancreatic β-cell function and insulin secretion [61]. Cortisol also shifts energy storage toward visceral adiposity, a key feature of metabolic syndrome and a significant risk factor for CVD [64].

Concurrent activation of the SNS leads to increased release of catecholamines (epinephrine and norepinephrine). Acute stress leads to elevations in catecholamines, while chronic distress results in sustained sympathetic tone, decreased vagal activity, and reduced HRV [5,6]. Catecholamines stimulate hepatic glucose production, inhibit insulin-mediated glucose uptake, and promote lipolysis, ultimately increasing circulating free fatty acids, which further impair insulin signaling (Figure 3) [41,63]. SNS activation also increases blood pressure, heart rate, and myocardial oxygen demand, which can precipitate arrhythmias and myocardial ischemia, particularly in individuals with underlying coronary artery disease (CAD) [41,63].

In parallel, stress-related renin-angiotensin-aldosterone system (RAAS) upregulation has also been observed in individuals with depressive symptoms, resulting in increased vascular tone, sodium retention, and adverse remodeling, reinforcing hypertension and cardiometabolic risk [65,66]. The AHA recognizes these neuroendocrine mechanisms as central to the mind-heart-body connection, noting that chronic psychological distress leads to increased sympathetic tone, decreased vagal tone, and impaired autonomic regulation, all of which are associated with increased arterial stiffness, endothelial dysfunction, and heightened cardiovascular risk [41].

Autonomic Dysregulation

Distress states (e.g., depression, PTSD) are characterized by heightened sympathetic drive and reduced parasympathetic (vagal) tone, reflected clinically as lower HRV and greater sympathetic predominance. Meta-analyses and data from the Framingham study demonstrated reduced HRV that predicts incident cardiovascular events and mortality independent of traditional risk factors [67-69].

In patients with established CAD, mental stress has also been shown to provoke myocardial ischemia in 15%-20% of patients [70]. “Mental stress-induced myocardial ischemia” (MSIMI), driven by autonomic-vascular dysregulation and microvascular constriction, promotes worse prognosis in coronary disease [71]. In a recent publication, Vaccarino et al. showed that MSIMI is associated with a 2.5-fold increase in the composite endpoint of cardiovascular death and first or recurrent nonfatal myocardial infarction [72].

Procoagulant State

Psychological distress, encompassing acute and chronic stress, depression, anxiety, and PTSD, is increasingly recognized as a driver of hypercoagulability, which is a key mechanistic link to CVD (Figure 1). Activation of the SNS and the HPA axis during psychological stress leads to increased release of catecholamines and cortisol, which in turn promotes platelet activation, upregulation of coagulation factors, and suppression of fibrinolysis [73,74]. Acute stress-induced hypercoagulability is initially adaptive, facilitating hemostasis in response to injury; however, it becomes maladaptive under chronic or excessive stress, thereby increasing the risk of arterial and venous thrombotic events (Figure 1) [75,76].

Prospective cohort studies and Mendelian randomization analyses suggest that stress-induced changes in hemostatic factors are partially causal for incident atherosclerotic CVD and venous thromboembolism [75]. Early studies have also demonstrated that acute episodes of anger or severe emotional stress may trigger acute coronary syndromes, and mental stress may enhance platelet activation in patients with angina and ischemic heart disease [77,78]. These demonstrated hemostatic shifts to human stress paradigms support a biologically plausible route by which mental distress can precipitate plaque thrombosis and acute coronary events.

Increased Inflammation

Inflammation is now recognized as a central mediator of plaque formation, destabilization, and rupture, with low-grade sterile inflammation involved in all steps of atherogenesis [79]. Acute psychological stress reliably triggers a significant increase in key circulating proinflammatory markers, creating a state of temporary systemic inflammation. A meta-analysis of laboratory stress challenges revealed consistent and time-dependent elevations in proinflammatory cytokines, including interleukin-6 (IL-6), interleukin-1β (IL-1β), and tumor necrosis factor-α (TNF-α) [80]. This inflammatory reactivity is believed to form a critical physiological link between exposure to environmental stressors and long-term health risks. Depression, a common manifestation of chronic distress, has also been shown to be associated with higher proinflammatory cytokines, including C-reactive protein and IL-6 [81].

These biomarkers reliably prospectively predict the risk of major adverse CVD events and CHD [82]. The biological rationale for this predictive power lies in the direct role these cytokines play in driving vascular inflammation, a key process in the pathogenesis of atherosclerosis. IL-6 and TNF-α are involved in both systemic inflammation and local tissue inflammation, promoting processes that can lead to plaque instability and rupture [32,82]. This link is further strengthened by clinical evidence showing that anti-inflammatory drugs targeting these pathways (e.g., canakinumab, which targets IL-1β and lowers IL-6) reduce cardiovascular event rates in secondary prevention trials [83].

Altered Immune Response

Chronic psychological stress disrupts neuroendocrine and immune regulation through the sustained activation of the SNS and HPA axis, resulting in excessive release of catecholamines and glucocorticoids [84]. These stress hormones alter immune cell function, creating a state of low-grade systemic inflammation while impairing innate and adaptive immunity, a mechanism that links stress with CVD (Figure 1), autoimmune disorders, and impaired wound healing. Psychological distress further exacerbates this dysregulation, enhancing proinflammatory cytokine production, altering leukocyte trafficking, and stimulating hematopoietic tissues, such as the bone marrow and spleen [41,49,79,85].

Animal models and human imaging studies demonstrate that stress-related activation of hematopoietic tissues and the amygdala worsens arterial wall inflammation, accelerates atherosclerosis, and increases circulating inflammatory cytokines [85]. Major depressive disorder exemplifies this process, with elevated IL-6 and TNF-α levels and markers of endothelial dysfunction such as soluble adhesion molecules and von Willebrand factor [86]. Impaired immune responses, increased sympathetic tone, reduced vagal tone, and decreased HRV are key pathways by which chronic psychological distress promotes arterial inflammation and atherogenesis, ultimately elevating the risk of CVD [41,85,86].

Increased Insulin Resistance

Over the past 25 years, the role of increased insulin resistance has become increasingly recognized as one of the key pathogenic factors in the development of T2DM, CVD, and metabolic syndrome [87,88]. However, when exploring the underlying causes of this increased insulin resistance, it becomes evident that it cannot be attributed to a single causative mechanism but rather to a constellation of contributing factors, among which increased stress hormones that is driven by chronic activation of the HPA axis and the SNS that plays a significant role, impacting insulin signaling and exacerbating metabolic dysfunction [89].

In 1996, Räikkönen et al. demonstrated that psychosocial stress, including excessive tiredness, type A personality, hostility, and anger, was independently associated with insulin resistance and related metabolic abnormalities, such as hyperinsulinemia, dyslipidemia, hypertension, abdominal obesity, and elevated plasminogen activator inhibitor-1. These associations remained significant after adjusting for potential confounders such as smoking, body mass index, age, education, physical activity, and alcohol consumption, thereby providing additional evidence that chronic stress contributes to the development of insulin resistance and metabolic syndrome [90].

In the setting of increased psychological distress, persistent activation of the HPA axis disrupts insulin signaling through increased serine phosphorylation of insulin receptor substrate-1 and downregulation of phosphatidylinositol 3-kinase, ultimately leading to reduced glucose transporter-4 expression in skeletal muscle and adipose tissue, thereby contributing to insulin resistance (Figure 3) [91].

Increased Oxidative Stress

Oxidative stress refers to a disruption in the balance between the generation of reactive oxygen species (ROS) and the body’s ability to neutralize them with antioxidant defenses [92]. It has been shown that subjects exposed to prolonged psychological stress consistently exhibit elevated levels of oxidative stress biomarkers (Figure 1), including lipid peroxidation products and oxidized nucleotides [93]. Chronic stress leads to sustained glucocorticoid elevation, which disrupts mitochondrial oxidative phosphorylation by impairing electron transport chain function, particularly at complexes I and III, thereby increasing electron leakage and superoxide generation, while concurrently downregulating key antioxidant enzymes such as glutathione peroxidase, superoxide dismutase, and catalase, ultimately diminishing the cell’s ability to neutralize reactive oxygen species and maintain redox balance [94].

While oxidative stress has been implicated in the pathogenesis of diabetes and insulin resistance [95], extensive research has elucidated that chronic hyperglycemia in T2DM induces persistent oxidative stress primarily via mitochondrial overproduction of reactive oxygen species (ROS), which initiates a spectrum of maladaptive molecular pathways, including flux through the polyol pathway, activation of the hexosamine biosynthetic route, upregulation of protein kinase C β isoforms, and accumulation of advanced glycation end products, culminating in impaired insulin signaling, endothelial dysfunction, and the acceleration of both microvascular and macrovascular diabetic [96].

Increased oxidative stress impacts numerous physiological systems in the body and also plays a role in enhancing low-density lipoprotein oxidation, thereby promoting atherosclerotic plaque formation [97]. Moreover, it is recognized as one of several mechanisms affecting the cardiovascular system; notably, elevated TNF-α levels have been shown to directly induce mitochondrial ROS production within cardiac myocytes [98].

Hemodynamic Dysregulation

Hemodynamic dysregulation caused by stress is directly linked to CVD through several well-established pathophysiological mechanisms (Figure 1). As discussed before, acute psychological stress activates the SNS and the HPA axis, resulting in surges of catecholamines and glucocorticoids that elevate heart rate, blood pressure, and myocardial oxygen demand. These changes can precipitate myocardial ischemia, arrhythmias, and even acute myocardial dysfunction such as Takotsubo cardiomyopathy, particularly in individuals with underlying CAD. Stress-induced autonomic dysregulation can perturb cardiac innervation, increase the risk of plaque rupture, and trigger acute coronary events [41].

MSIMI, as mentioned earlier, is a recognized phenomenon that often occurs at lower cardiac workloads than exercise-induced ischemia and is primarily mediated by paradoxical coronary vasoconstriction and microvascular dysfunction [79,99]. Chronic stress further contributes to the development and progression of CVD by sustaining increased sympathetic tone, reducing vagal activity, and promoting endothelial dysfunction and arterial stiffness. This persistent imbalance accelerates atherosclerosis, increases the risk of hypertension, and impairs baroreflex sensitivity, thereby elevating long-term cardiovascular risk [79,100]. Epidemiological data demonstrate that both acute and chronic stress exposures are independent risk factors for incident CAD, stroke, and cardiovascular mortality, even after adjustment for traditional risk factors [101].

Therapeutic intervention for stress reduction: implications for diabetes, hypertension, and CVD

Various interventional strategies have been implemented to reduce stress in various populations, including working adults. College students, medical students, first responders, as well as patients with various illnesses ranging from diabetes, hypertension, CVD, and cancer [5,102-115], in addition to special populations such as intensive care unit-related psychological stress, are increasingly recognized entities with serious consequences that largely go undetected and untreated [106,116-118].

Several nonpharmacological interventions have been proven successful in psychological distress reduction [105,106], including Mindfulness-based interventions (MBIs), mindfulness-based group intervention [102], mind/body intervention [103], and holistic lifestyle mobile health intervention [115], among others. These interventions have been implemented in various research protocols, including community-based controlled trials [9] and randomized controlled trials [108,111]. These, in addition to various meta-analyses documenting the effectiveness of these interventional studies [102,104], provide strong evidence.

Therapeutic interventions have been shown to be effective even in the short-term trials; for example, in a meta-analysis aimed to assess the effectiveness of MBIs for reducing psychological distress in working adults that involved 1,139 participants, effectiveness was largely maintained at a median follow-up of five weeks with data suggesting that brief versions of mindfulness-based stress reduction developed for organizational settings are equally effective as standard eight-week versions originally developed for clinical settings [102].

Stress reduction strategies have been shown to improve glycemic control and outcomes in the diabetic population [110,112-114], with ongoing studies aimed at the prevention of type 2 diabetes among at-risk populations such as women with gestational diabetes (GDM) [115]. In an ongoing study of a one-year randomized controlled trial (RCT) with a three-year follow-up period involving post-GDM women with no current diabetes diagnosis, participants were randomized to either an Intervention group, including a smartphone-based, conversational agent-delivered holistic lifestyle, or a comparison group [115].

Women from both groups will be provided with an Oura ring for tracking physical activity, sleep, and HRV, among other measures. Outcomes of the study include measurements of the effects of these interventions on body mass index and blood pressure, as well as results from oral glucose tolerance tests [115]. This important trial, utilizing the state-of-the-art technology and taking advantage of the mobile phone accessibility, is bound to provide crucial information regarding the effectiveness of stress management in the prevention of type 2 diabetes, among women with GDM, a very high-risk population with nearly a 12-fold increased risk for developing overt diabetes in four to six years post-GDM.

Stress management strategies have been shown to be highly effective in the management and prevention of CVD with improved outcomes, particularly among vulnerable populations such as women [119-125], where control of CVD risk factors appears to be largely suboptimal [126].

Collectively, these interventions of stress reduction that are feasible in the community and clinical practice settings support the premise that psychological distress is manageable and can lead to the reduction of diabetes and control of other CVD risk factors.

## Conclusions

In this review, we presented the current evidence from both cross-sectional and longitudinal studies demonstrating strong associations between psychological distress, diabetes, and CVD. We also enumerated the various scales used in different studies to assess and identify psychological distress. We highlighted, in depth, the underlying pathophysiologic mechanisms of CVD and insulin resistance/diabetes that are operative in the states of psychological distress, including the gender differences in the interplay between psychological distress and CVD.

Finally, we discussed the current therapeutic interventions that have been successful in reducing psychological distress in various clinical settings. Thereby, we conclude that psychological distress is a viable and potentially modifiable therapeutic target that is feasible to address in various healthcare settings to reduce CVD and diabetes risk, particularly among high-risk populations.
